# Determinants of Russian Attitudes Toward a Green Economy During the Covid-19 Pandemic

**DOI:** 10.11621/pir.2023.0107

**Published:** 2023-03-30

**Authors:** Aleksandr A. Maksimenko, Olga S. Deyneka, Ekaterina V. Zabelina

**Affiliations:** a HSE University, Moscow, Russia; b St. Petersburg University, Russia; c Chelyabinsk State University, Russia

**Keywords:** Attitudes toward a green economy, COVID-19 pandemic, the “Green Economy” questionnaire, environmental attitudes, pro-environmental behavior

## Abstract

**Background:**

The development of environmental consciousness is a necessary part of the full development of society. The pandemic caused by COVID-19 has increased attention to the problems of man’s relationship with nature, and the green behavior of both the consumer and the producer. Attitudes toward a green economy are especially important to study in countries rich in natural resources, as they have more opportunities to overcome the contradictions between economic growth and green innovation.

**Objective:**

The purpose of this study was to identify the determinants of Russian attitudes toward a green economy during the COVID-19 pandemic. The main hypothesis was that demographic factors determine attitudes toward a green economy in different ways, including the willingness to take actions in support of it, and acknowledgement of the connection of the need for green transformations with the pandemic.

**Design:**

Subjects were given the questionnaire “Green Economy” which contains 19 statements with which they needed to express their degree of agreement on a 5-point Likert scale. Potential determinants of their attitudes toward a green economy were collected using an additional questionnaire, which included indicators of gender, age, family and professional status, religiosity, income level, education level, and place of residence (locality). The study involved 874 respondents from the Russian Federation (62.4% female; 37.6% male; the average age was 37.34 years).

**Results:**

The results of a regression analysis showed that women, people with increased religiosity (but not too religious), younger people, and students and employees of public organizations (as opposed to employees of state and commercial organizations), as well as people from small towns or rural areas, were more positive about the idea of transition to a green economy.

**Conclusion:**

The belief that the pandemic situation has reinforced the need for a transition to a green economy was influenced by gender, degree of religiosity, and place of residence. Women, to a greater extent than men, as well as people who were more religious and lived in small towns and rural areas, were more acutely aware of the impact of the pandemic on the actualization of environmental problems.

## Introduction

The increase in emissions polluting the environment, climate change, and other global environmental problems make the issue of transition to a green economy extremely topical. The term “green” connotes a new look at the development of human wellbeing through the construction of a new ecological civilization in the world (Liao, 2017). Historical responsibility for the state of the environment is a criterion for self-consciousness, reflecting the “maturity” of such a civilization (Valkovskaya, 2017, p. 219). Researchers call for social and environmental responsibility by business, based on the concept of sustainable development and the need for implementation of a consistent state environmental policy (Uskova & Kopytova, 2018) to strengthen institutional support for the transition to a green economy (Kozhevnikov & Lebedeva, 2019). Some scholars believe that the main objective of a “green” economy is not the greening of the technological base, but the greening of consciousness, both of the population (Khaliy, 2015), and, above all, of specialists, managers, and authorities (Lebedev, 2015; Thirnadtsatko, 2022).

Although there is no single definition of a “green” economy, this paradigm usually reflects three goals: improving resource efficiency; ensuring ecosystem sustainability; and strengthening social justice (Speck & Zoboli, 2017).

Despite the urgency of the need to create a green economy, many scientists question the economic efficiency of such a transition and its feasibility (Schmalensee, 2012; Ehresman & Okereke, 2015; Barrier, 2016). Their arguments include: 1) violations of green laws by the elite (Montefrio & Dressler, 2016); 2) data on the use of resources and carbon emissions (Hickel & Kallis, 2020); 3) low wages and little social protection for jobs created in the field of alternative energy (Chen & Li, 2021) and other negative social consequences (Dauvergne & LeBaron, 2013); 4) low productivity in green economy sectors in the cities with low per capita income (Wang, Hu, & Li, 2021); 5) reduced productivity in the industries with an increase in the share of “green” employment (Elliott & Lindley, 2017); 6) unwillingness to reduce consumption against the background of technical and organizational progress (Sanne, 2020); 7) “double dividends” from green taxation in the presence of unemployment (Fitz-Roy & Smith, 2004); and 8) the risk of increasing poverty (Rutskiy & Filippov, 2022), in particular in agricultural areas (Barbier, 2016).

Many factors influencing the creation of a green economy are being studied: namely, environmental innovations (Speck & Zoboli, 2017); open circulation of “green” knowledge (Speck & Zoboli, 2017; Guo et al., 2014); availability of financial resources for long-term investment (Speck & Zoboli, 2017; Winnett & Lewis, 2020); fiscal reforms (Speck & Zoboli, 2017); legislative changes such as introduction of “green” public procurement (Diofasi-Kovacs & Valko, 2017; Pasnicu & Ciuca, 2020); and green taxes (Sanne, 2020). At the same time, insufficient attention is being paid to psychological factors in this area, such as people’s attitudes toward a green economy and willingness to support it with real actions. Despite the fact that some scientists call for changing people’s thinking in order to achieve a “green” economy (*e.g.,* Speck & Zoboli, 2017), there are few real studies of such factors.

In addition, there is some thought that the economic problems caused by the COVID-19 pandemic have exacerbated the existing contradictions in the green sphere (Streimikiene & Kaftan, 2021; Chen et al., 2020; Kaur & Kaur, 2021; Lahcen et al., 2020; Goenka, Liu & Nguyen, 2021; Rutskiy & Filippov, 2022). Some researchers consider the pandemic as a condition and incentive for the development of a green economy (Gawel & Lehmann 2020; Barbier, 2020); others warn about its negative impact on the environment due to the contradiction between the objectives of economic growth (the effect of higher productivity and greater labor supply) and the principles of a green economy (Lahcen et al., 2020; Goenka, Liu, & Nguyen, 2021). However, the attitude of the population toward this issue is still insufficiently studied. In particular, we agree with those who note the lack of research on the impact of Covid-19 on environmental consciousness and behavior, both in Russia and abroad (Mdivani & Alexandrova, 2021).

The purpose of this study was to identify the determinants of attitudes toward a green economy during the COVID-19 pandemic in the Russian population. Our main hypothesis was that demographic factors would determine attitudes toward a green economy in different ways, including the willingness to take actions in support of it, as well as acknowledging the connection of the need for green transformations with the pandemic.

## Methods and sample

The “Green Economy” questionnaire was used as our main tool. The questionnaire is based on 17 Sustainable Development Goals, which would form new requirements for the key components of a green economic system: production, exchange, distribution, and consumption. These statements are supplemented by expressions about the relationship between the green economy and the pandemic. The questionnaire contains 19 statements with which respondents needed to express their degree of agreement on a 5-point Likert scale (1 = “I do not agree at all; 2 = “I rather disagree”; 3 = “I find it difficult to answer”; 4 = “I rather agree”; 5 = “I completely agree”).

Potential determinants of attitudes towards the green economy were collected using another questionnaire survey, which collected indicators of gender, age, family and professional status, religiosity, income level, education level, and place of residence (locality) of the participants.

To identify the contribution of the demographic indicators to the formation of a positive attitude toward a green economy, regression analysis was used. To compare the intensity of the attitudes toward a green economy in different categories of the population, a one-factor analysis of variance was used.

The study involved 874 respondents from the Russian Federation, 62.4% female, and 37.6% male. The age of the respondents ranged from 15 to 77 years, while the average age was 37.34 years. Subjectively, 0.9% of the respondents rated their income as “very high,” 3% as high, 56.3% as average, 33.0% as low, and 6.9% as very low. When asked about religiosity, 1.5% of the respondents considered themselves very religious, 30.3% quite religious, 45.5% not very religious, and 22.7% not religious at all. As to the level of education, 18.4% of the respondents had a secondary education, 14.3% an incomplete higher education, and 44.1% higher education (bachelor’s or specialist’s degree). Additionally, 16.6% had completed a master’s degree, and 6.6% had a scientific degree. The marital status of the respondents was: 46.5% officially married, 11.7% living with a partner, 10.6% divorced, 27.8% single, 2.5% widowed, and 0.9% married but living separately.

## Results

**Figure 1.**
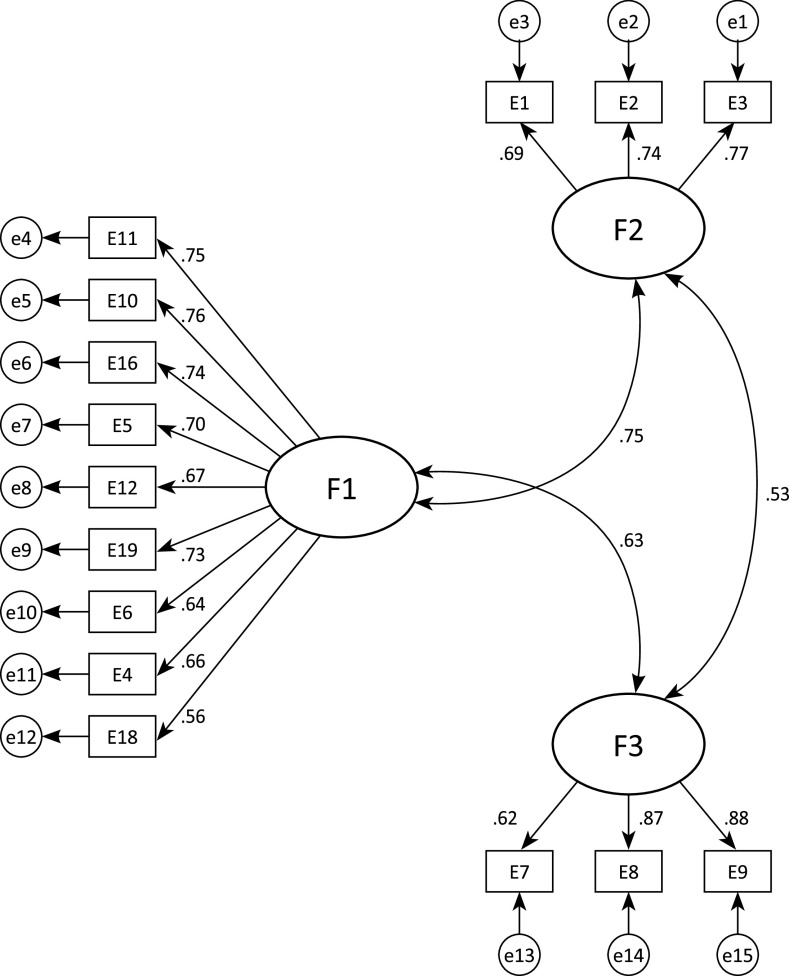
Structural model of the questionnaire based on the results of confirmatory analysis

*Note. F— factors, E— variances of agreement with the statements of the questionnaire, e— errors of variables*

The confirmatory factor analysis resulted in the final (a posteriori) model of the respondents’ attitudes toward a green economy, which consisted of three factors: positive attitudes toward a green economy, the connection of a green economy with the pandemic, and support for a green economy with real actions (*Figure 1*). The model corresponded well to the initial data on the indicators of the indices: CMIN = 545.308; df = 87; p = 0.000; GFI = 0.928; CFI = 0.927; RMSEA = 0.078. The estimated parameters of the model turned out to be statistically reliable: regression coefficients (p < 0.001); variance of latent variables (scales) (p < 0.001); and covariances (correlations) between errors (p < 0.01). Correlations between all the scales also showed statistical validity (p < 0.01).

The first factor, which was the most significant and defined as “Positive attitudes toward the green economy,” combined the variance of agreement with the following numbered statements: “I support the principle of a green economy, according to which natural resources should go to our descendants” (No. 11);“I am convinced that the concept of a green economy supports the conservation of resources and the reduction of negative impacts on nature” (No. 10); “I believe the transition to a green economy is morally correct” (No. 16); “Countries should introduce coefficients that evaluate overall well-being based not only on monetary income and expenditure, but also on energy and environmental factors” (No. 5); “I approve the introduction of an international system for assessing natural and human capital in enterprises” (No. 12);“I believe that the costs of introducing green technologies will pay off in the future” (No. 19);“I am ready by my actions to support public initiatives aimed at preventing a pandemic and other global threats” (No. 6);“Businesses should start adopting green technologies, regardless of their cost” (No. 4); and “In order to survive during a pandemic, humanity will have to unite and learn to negotiate in the face of a common danger.” (No. 18)

The second factor, called the “Connection of the green economy with the pandemic.” included responses to the following statements: “During and after the pandemic, companies need to move from focusing only on profit to caring also about the environment” (No. 1); “The only alternative to the development of the economy against the backdrop of a pandemic is an environmentally oriented economy” (No. 2); and “The pandemic should give impetus to increased investment in the environment, environmental research, and technology.” (No. 3)

The third factor, labeled “Support for the green economy by real actions,” included the variance for the following statements: “I am ready to sign public appeals on the Internet aimed at preventing a pandemic, drawing attention to climate change, introducing green technologies” (No. 7); “I am ready to donate money for the conservation of nature, if I am sure that it will be used for their intended purpose” (No. 8); and “I am ready to donate money to funds whose main goal is to prevent pandemics, combat environmental pollution, and preserve biodiversity on the planet.” (No. 9)

It should be noted that some of the statements did not gain the necessary weight to be included in the analysis, so they were excluded. They were: (No. 13) “I have doubts that a ‘green’ economy will contribute to the eradication of poverty, increase the country’s competitiveness and growth in key sectors;”(No. 14)“A ‘green’ economy is a socially just economy;”(No. 15)“I have confidence in the implementation of the principles of a green economy;” and (No. 17) “I have never worried about global risks (such as global epidemics, climate change, depletion of world resources, etc.”). We assumed that the reason lay in the formulation of the statements. For example, statements 13 and 17 are formulated in a negative form and could be less clear to the respondents. Statements 14 and 15 are probably too idealized; they touch on such phenomena as trust and justice, which could be hard to assess.

On checking the reliability of the scales based on the internal consistency of their statements, the Cronbach’s a coefficients showed acceptable levels of reliability: “Positive attitudes towards the green economy” (α = 0.890); “The connection of the green economy with the pandemic” (α = 0.778); and “Support for the green economy with real actions” (α = 0.819).

Next, we examined which demographic variables might act as determinants of the participants’ attitudes toward a green economy. To do this, the value of each scale was calculated, and was then added as an independent variable to the regression analysis.

The results of the regression analysis showed that variables of gender, religiosity, age, professional sphere, and place of residence (type of settlement) contributed to a positive attitude towards the green economy (R = .238; R2 = .057) (See *Table 1*).

**Table 1:** Regression coefficients: dependent variable - positive attitude toward a green economy

	Model	Non–standardized coefficients		Standardized coefficients	t	p
		B	Standard error	Beta		
1	(Constant)	3.933	.030		130.183	.000
	Gender	–.248	.049	–.168	–5.037	.000
2	(Constant)	4.223	.094		44.799	.000
	Gender	–.238	.049	–.161	–4.838	.000
	Religion	–.102	.031	–.108	–3.249	.001
3	(Constant)	4.395	.114		38.413	.000
	Gender	–.224	.049	–.152	–4.550	.000
	Religion	–.098	.031	–.104	–3.121	.002
	Age	–.005	.002	–.088	–2.636	.009
4	(Constant)	4.316	.121		35.713	.000
	Gender	–.243	.050	–.165	–4.852	.000
	Religion	–.101	.031	–.108	–3.246	.001
	Age	–.005	.002	–.090	–2.693	.007
	Occupation	.048	.024	.068	2.003	.045
5	(Constant)	4.110	.156		26.346	.000
	Gender	–.235	.050	–.159	–4.682	.000
	Religion	–.101	.031	–.108	–3.251	.001
	Age	–.005	.002	–.078	–2.316	.021
	Occupation	.053	.024	.075	2.210	.027
	Place of residence	.062	.030	.070	2.080	.038

The results of the regression analysis showed that women, people with increased religiosity (but not too religious), younger people, and students and employees of public organizations (as opposed to employees of state and commercial organizations), as well as people from small towns or rural areas, were the most positive about the idea of transition to a green economy.

Next, we explored the determinants of the belief in the connection between the green economy and the pandemic caused by the coronavirus (*Table 2).*

**Table 2:** Regression coefficients: dependent variable — the relationship between the pandemic and a green economy

	Model	Non–standardized coefficients		Standardized coefficients	t	p
		B	Standard error	Beta		
1	(Constant)	3.666	.037		99.114	.000
	Gender	–.329	.060	–.181	–5.446	.000
2	(Constant)	4.083	.115		35.457	.000
	Gender	–.313	.060	–.173	–5.223	.000
	Religion	–.146	.038	–.127	–3.821	.000
3	(Constant)	3.884	.153		25.401	.000
	Gender	–.300	.060	–.165	–4.968	.000
	Religion	–.145	.038	–.126	–3.796	.000
	Place of residence	.071	.036	.065	1.970	.049

The belief that the pandemic situation reinforced the need for a transition to a green economy was influenced by gender, degree of religiosity, and place of residence. Women, to a greater extent than men, as well as people who were more religious and lived in small towns and rural areas, were more acutely aware of the impact of the pandemic on the actualization of environmental problems (R = .230; R2 = .053).

Next, we studied which variables influenced people’s willingness to support the principles of a green economy with real actions (*Table 3*).

**Table 3:** Regression coefficients: dependent variable — willingness to act in support of a green economy

	Model	Non–standardized coefficients		Standardized coefficients	t	p
		B	Standard error	Beta		
1	(Constant)	3.804	.108		35.338	.000
	Age	–.014	.003	–.173	–5.180	.000
2	(Constant)	4.288	.162		26.424	.000
	Age	–.014	.003	–.165	–4.986	.000
	Religion	–.175	.044	–.131	–3.962	.000
3	(Constant)	4.152	.171		24.225	.000
	Age	–.014	.003	–.169	–5.105	.000
	Religion	–.183	.044	–.137	–4.137	.000
	Occupation	.080	.033	.080	2.405	.016
4	(Constant)	4.144	.171		24.265	.000
	Age	–.013	.003	–.160	–4.831	.000
	Religion	–.177	.044	–.133	–4.018	.000
	Occupation	.098	.034	.097	2.883	.004
	Gender	–.193	.071	–.092	–2.733	.006

According to the results of the regression analysis, the willingness to take real actions in support of a green economy depended on age, level of religiosity, professional status, and gender (R = .248; R2 = .061). Younger people with increased religiosity, mostly women, as well as students and employees of public organizations (as opposed to employees of state and commercial organizations), were the most willing to act in support of a green economy.

Thus, the main determinants predicting a positive attitude towards the green economy were female gender, younger age (students), and a high (but not very high) level of religiosity, as well as the relatively small size of place of residence, and belonging to a public organization. Variables such as education level, family status, and income level did not directly affect the shaping of a positive attitude towards a green economy. The main hypothesis was partially confirmed: the level of religiosity was a constant predictor of all measured parameters, while gender, age, place of residence, and type of organizational affiliation varied.

## Discussion

Analyzing the data obtained from the point of view of compliance with previous studies, we found that the determinants we identified largely corresponded to the factors of environmental attitudes and pro-environmental behavior they found. In particular, our results were consistent with data on the influence of gender, age (Emelyanova, Nestik, & Belyh, 2019), and religiosity (Ahmad et al., 2012) on environmental attitudes and behavior. Our results do not contradict the results of other authors on the relationship between environmental consciousness and the respondents’ ideas about Covid-19 (Mdivani & Alexandrova, 2021), which indicated that the attention of the younger generation is more focused on the consequences of the human impact on nature (ecocentric type), while representatives of the older generation are more concerned about the negative impact of nature on humans (archaic type).

Our study allows us to make two important generalizations. First, the fact that the pandemic has exacerbated the need for a transition to a green economy was confirmed not only by external (economic) calculations, but also by internal factors. In the minds of many people, the pandemic and the issue of establishing a green economy appeared related. Such a relationship leads to the civilizational issue of preserving oneself as a biological species (homo sapiens) in the face of violations of the ecological balance of the planet caused by our species (Panov, 2017).

Second, the fact that the willingness to take real actions in support of the green economy was a separate, relatively small factor may point to a gap between the declared belief in the correctness of green principles, and the willingness to implement them. This fact can become a powerful deterrent force in the implementation of green reforms. Other authors have also noted the insufficient activity of Russians in matters of environmental protection (Dushkova & Kirilov, 2017), as well as a low level of ecological culture, which is distinguished by fragmentation, distance, and inconsistency of its components (Kurbanov & Prokhoda, 2019).

Therefore, when solving such a strategic task as improving environmental education and enlightenment (Dukhanina & Maksimenko, 2019), it is important to combine the transmission of knowledge with the development of skills, combining information with involvement in pro-environmental behavior as expressed in the theory of pushing (Thaler & Sunstein, 2008). Environmental education also involves the development of spiritual activity and moral consciousness. In scientific research, it is advisable to strengthen the general tendency to integrate the solution of practical problems with the need for theoretical understanding of the constructs and models of environmental behavior (Morozova & Kozlov, 2020). To solve environmental problems in practice, it is important to remove the contradictions between the declared goals of environmental and economic development stated in the legal documents and the lack of development of institutional support for their implementation (Kozhevnikov & Lebedeva, 2019).

## Conclusion

The results of our study enrich science with the latest data on the determinants of Russian attitudes toward a green economy during the COVID-19 pandemic, and also indicate an increase in pro-environmental sentiment in connection with the epidemic. The study’s findings about the influence of gender, age, level of religiosity, the size of the residential location, and the type of work organization in the shaping of a positive attitude toward a green economy and willingness to act in its support contribute to the development of environmental psychology.

The data obtained should be taken into account when developing state programs for the transition to a green economy. In particular, it is necessary to address a certain inertia of the population in taking concrete steps along this path. The main driver for dealing with this issue may be the younger generation, especially the female part of it, which shows itself to be more sensitive to environmental problems. Increasing the level of religiosity of the population can also become a key factor involved in the green transformation. On the contrary, an increase in atheistic sentiments in the society can become a barrier to the introduction of green technologies. The transition to a green economy is easier to implement in small towns and villages, where people perceive its basic principles more positively and readily.

Our study contributes to the development of the ideas about the factors influencing the transition to a green economy in a country with an emerging economy, with a focus on some demographic variables as predictors of the positive attitude toward a green economy.

The main limitations of the study included the use of the survey methods only, which may not provide completely objective data. The results can be extrapolated to countries with growing economics, but can be applied with limitations to the countries with developing and developed economics. In addition, the results could have been influenced by the factor of the economic culture of the country, so in the future it is necessary to supplement the study with facts from other countries (cultures).
